# Impact of Language Barriers in Doctor – Patient Relationship: A Qualitative Study

**DOI:** 10.12669/pjms.39.1.5805

**Published:** 2023

**Authors:** Remsha Mustafa, Usman Mahboob, Rehan Ahmed Khan, Abeer Anjum

**Affiliations:** 1 Dr. Remsha Mustafa, MBBS, MHPE. Assistant Professor, Department of Medical Education, Khawaja Muhammad Safdar Medical College, Sialkot, Pakistan; 2 Dr. Usman Mahboob, MBBS, MPH, FHEA, DHPE, Fellow FAIMER. Associate Professor, Institute of Health Professions Education & Research, Khyber Medical University, Peshawar, Pakistan; 3 Dr. Rehan Ahmed Khan, MBBS, FCPS, FRCS, JM-HPE, Ph.D. Assistant Dean of Medical Education, Professor of Surgery, Islamic International Medical College, Riphah International University, Rawalpindi, Pakistan; 4 Dr. Abeer Anjum, MBBS, MHPE. Assistant Professor, Department of Medical Education, Khawaja Muhammad Safdar Medical College, Sialkot, Pakistan

**Keywords:** Culture, Recent medical graduates, Language barriers, Language discordance, Effective Doctor-patient relationship

## Abstract

**Objective:**

To explore the problems faced by recent medical graduates in communication with their patients due to language barriers and the influence of these language barriers on the doctor-patient relationship.

**Methods:**

A basic qualitative study was conducted at Allama Iqbal Memorial Teaching Hospital Sialkot, Services Hospital Lahore and Mayo Hospital Lahore over eight months after receiving approval from the Ethical Review Board of the University of Lahore. Twelve recent medical graduates from different departments were selected via a purposive sampling technique. Data was collected through semi-structured interviews conducted over the phone with prior appointments. Manual qualitative thematic analysis was done by transcribing the interview and then codes, subthemes, and themes were generated.

**Results:**

Six themes and thirteen subthemes were identified depicting the influence of language barriers on the doctor-patient relationship, namely: frustration (due to repetition, feeling of inadequacy & disappointment), lack of rapport (difficulty in communication, effective counseling & failure in establishment of comfort level), trust Issues (predilection towards the native speaker & difficulty in getting consent) patient dissatisfaction, compliance issues (difficulty in comprehending medication & nature of disease), and threat to patient safety (misdiagnosis & consequent treatment plan & misinterpretation of treatment)

**Conclusion:**

This study establishes the detrimental effects of language barriers on the relationship between physician and patient which can help medical educationists and policymakers in devising a curriculum in such a way that it can minimize the impact of language barriers on the doctor-patient relationship.

## INTRODUCTION

Pakistan is a land of diverse cultures and languages. Punjabi is the most widely spoken language in Pakistan; whereas only 7.57% of the people of Pakistan speak Urdu, as their first language.^1^ Although Urdu alongside English shares the status of the official language still, most of the people living in rural areas do not comprehend either of the languages.^2^

The curriculum and medium of instruction used in all medical colleges and universities throughout Pakistan are English^3^ with not even a single course in the curriculum of Bachelor of Medicine and Surgery (MBBS) throughout the five years that focuses on the translation of common signs and symptoms, patient complaints and diseases into the local or national language. Teaching doctor-patient communication is also neglected in many medical schools. Whereas, most patients visiting teaching hospitals and tertiary care units belong to diverse backgrounds and speak different languages. A local study conducted at two hospitals in Lahore identified a lack of language proficiency, different accent, use of medical jargon, non-verbal cues, acronyms, and inauthentic translators as a few of the linguistic barriers perceived by doctors and patients during communication.^4^ Even if one goes through all the books on clinical presentations and methods, s/he is bound to get surprised when patients try to explain their symptoms in their native lingo. Linguistic barriers are endorsed as a hindrance to effective doctor-patient communication in several studies.^5^,^6^ Language barriers between doctor-patient contribute to poorer patient assessment, misdiagnosis, increased adverse events, incomplete understanding of the patient condition and prescribed treatment, and impaired confidence in services received, thus poor quality of healthcare.^7^ Thus, doctors should be able to effectively communicate with patients for the provision of quality healthcare.^8^,^9^

To our knowledge, little research has been done in exploring the influence of language barriers on the doctor-patient relationship in Pakistan. Therefore, the objective of the study was to explore the problems faced by recent medical graduates in communication with their patients due to language barriers and the influence of these language barriers on the doctor-patient relationship.

## METHODS

A basic qualitative study was conducted at Allama Iqbal Memorial Teaching Hospital, Sialkot; Services Hospital, Lahore; Mayo Hospital, Lahore over eight months after receiving approval from the Ethical Review Board of University of Lahore (Ref. No. ERC/12/19/04, Dated: December 30, 2019).

### Population:

The target population included all the recent medical graduates (RMGs) of the MBBS program working in different hospitals in Punjab (mainly Lahore & Sialkot). We chose recent medical graduates as they are more prone to misunderstand their patients because during the five years of MBBS not even a single course addresses the clinical presentation of patients in their native language and also, they lack the experience required to understand the patient’s signs and symptoms when expressed in the native or national language. A purposive sample of twelve RMGs was selected who have earned their degrees not more than one year ago and have been working in their respective institutes for more than four months.

### Data Collection:

Data was collected through telephonic, semi-structured interviews in which a set of open-ended questions were asked from the participants regarding the issues they face due to ***language barriers when communicating with the patients and their influence on the doctor-patient*** relationship. Each interview lasted for 30-40 minutes. The interview questions were validated by six medical education experts and then piloted with one RMG to ensure clarity. Informed consent was taken and the appointment for the interview was fixed according to the availability of the interviewees. The place (Department of Anatomy, KMSMC) and the devices for the interview recording were the same and fixed for every interviewee and only a single and same researcher (first author) conducted all the interviews at the same place with the same devices. The interviews were audio-recorded on two separate devices to avoid any unforeseen circumstances. Notes were also taken. Due to the sensitivity of the interview questions, confidentiality and anonymity were maintained.

### Data Analysis:

Manual thematic analysis was done. All the audiotaped data was manually transcribed on the same day and counter-checked by the first author and then sent to the second author. Transcripts were read numerous times to get familiarized with the data. Inductive coding was done through a first and second cycle of coding. Both semantic and latent codes were identified. The codes were then merged to form subthemes and themes. The first and second authors analyzed the data independently, and the codes and themes were discussed and agreed upon to ensure analytical triangulation.

## RESULTS

Participants of our study were mainly from the department of medicine (41.7%) and the majority were females (75%). To maintain confidentiality, the names and identities of the participants were concealed. Each participant was allotted a number according to the number of interviews. Four participants were interviewed from each institute, Allama Iqbal Memorial Teaching Hospital, Sialkot; Services Hospital, Lahore; & Mayo Hospital, Lahore. Participants of the study belonged to the department of General Medicine (5;41.7%), Pediatrics (1;8.3%), General Surgery (4;33.3%), and Opthalmology (2;16.7%). Furthermore, participants reported Punjabi and Urdu as the most common languages used by the patient to communicate with doctors in these hospitals. Urdu was reported as the language used by all the participants in replying to the patients.

Six themes and thirteen subthemes were identified from data analysis that depicts the influence of language barriers on the doctor-patient relationship (Table-I).

**Table-I:** Influence of language barriers on the doctor-patient relationship

Themes	Subthemes	Representative Quote
Frustration	Negative impact on the doctor-patient relationship due to repetition	“They wouldn’t understand Urdu so making them understand their treatment and counseling them is really difficult and I pretty much get frustrated when I have to repeat myself again and again and even then the patient won’t understand it. So I think it can have a negative impact on t patient-doctor relationship.” (Dr.P3)
	Feeling of inadequacy and sadness	“Language barrier has caused intense frustration. I feel inadequate and sad for not being able to understand my patients and that results in avoidance and resentment.” (Dr.P7)
	Disappointment	“So what happens is that whenever any other doctor comes and he speaks in Punjabi, patients tell much more about themselves and the disease, their symptoms to that doctor rather than me….This is very much disappointing and frustrating.” (Dr.P1)
Lack of Rapport	Difficulty in communication	“I sometimes experience problems understanding the language people talk in especially Punjabi and I find difficulty understanding their accents. While taking history and doing examinations patients generally do not understand my command and I have to repeat it many times.” (Dr.P2).
	Difficulty in effective counseling	“If the patient belongs to a village and is Punjabi speaking and I am explaining everything to him/her in Urdu then the counseling will not be effective at all.”(Dr.P6)
	Failure in the establishment of comfort level	“Rapport building is very much affected by language because I think this is…ah this is a very basic thing which makes patients either comfortable or uncomfortable.” (Dr.P1)
Trust Issues	Predilection toward the native speaker	“I observed that patients were unable to discuss their medical issues with me and prefer other on-duty doctors.” (Dr.P7)
	Difficulty in getting consent	“… I explained the procedure…and I wrote the consent form and I asked them that do they want us to pass the double lumen but they immediately said no….. And when my senior PGR, he came and explained the procedure in the Punjabi language…Same things which I have told them, they immediately said yes for the language. So I think that was kind off I don’t know maybe it was a language barrier or trust issues.” (Dr.P1)
Patient Dissatisfaction	Difficulty to satisfy patient	“I am Urdu speaking and I do not have a grip on Punjabi…I will understand half of the things and half of the things I will not understand. Obviously, when I will not reply and explain to the patient adequately in their way. Patient will obviously think that the doctor is not good enough or be satisfied” (Dr. P5)
Compliance Issues	Difficulty in comprehending medication	“Once I was explaining the discharge medicine to a female attendant with a patient. I was explaining how to take it, the duration, and dose. After all that I have explained her, I asked her what have I told you. And she did not know anything.” (Dr.P11)
	Difficulty in comprehending the nature of the disease	“7 years old, known case of DKA just skipped insulin dose from 2 days. Due to language barriers, the patient’s mother was not aware with the nature of disease. She was casually saying that my son only skipped insulin from 2 days.” (Dr.P7)
Threat to Patient Safety	Misdiagnosis & consequent treatment plan	Today a patient came who was speaking simple Punjabi words but his accent was a bit different that’s why I couldn’t understand him. I faced a lot of difficulty in communicating with him but at the end whatever I understood I just wrote it.” (Dr.P3)
	Misinterpretation of treatment	“I remember once a patient came to me and I wrote 1 plus 1 on the prescription, it means that you have to take the 1 tablet 2 times a day. What patient understood was that he has to take 2 tablets at one time.” (Dr.P6)

## DISCUSSION

Our study highlights the problems faced by RMGs due to language barriers and their impact on the doctor-patient relationship. The findings of our study were consistent with that of previous literature.

Current results show that physicians get frustrated when patients fail to understand them. Literature review reveals that physicians frequently experience frustration, feeling of inadequacy, intimidation, and lack of confidence during their encounters with patients due to a lack of understanding resulting from the difference in language.^10^,^11^ Physician frustration can inhibit the delivery of quality healthcare to the patients, add to physician misery and lead to decreased follow-up referrals of patients, thus compromising the provision of healthcare to the patients.^10^,^12^

Our results report a lack of rapport due to language barriers as a consequence of the difficulty in communication, effective counseling, and failure of establishment of comfort level with patients. Rapport building via the use of empathy and effective communication skills is critical to forming effective and trusting relationships with patients and is essential to successful health outcomes.^13^,^14^ Whereas, lack of rapport has been associated with poor patient satisfaction, patient compliance, and healthcare outcomes in the general literature on the doctor-patient relationship.^13^

The current study highlights trust issues due to language barriers between physicians and patients. Our findings are supported by former literature that patient did not trust their physicians due to language barriers.^15^,^16^ Greater interpersonal trust between patient and physician is a foundation on which their relationship is built.^17^ Moreover, it has been found to improve patients’ confidence that the healthcare professionals have positive intentions,^18^ and loyalty to the physician.^19^ In addition, explicit discussion of therapeutic options is a central part of shared decision-making which relies on rapport and trust in the clinical setting.^20^ Whereas, distrust in physicians is a stumbling block to communication as the patient may not share important information or ask important questions.^17^

Another highly documented theme that appeared in the literature and is consistent with the results of our study was the lack of satisfaction with the healthcare system including their physician due to language barriers.^7^ It was cited in an article by Um and Lau that dissatisfaction among patients is associated with higher mortality rates and poor treatment outcomes.^21^ Furthermore, dissatisfied patients actively engage in negative word of mouth, are less loyal, and have an intent not to return to the healthcare service providers.^21^ Conversely, patient satisfaction is linked with patient compliance, loyalty to the physician, increased referrals, and enhanced health outcomes.^22^

One of the emerging themes was poor compliance with physicians’ advice and treatment. The results of our study of difficulty in comprehending medication and the nature of the disease are similar to those reported in the literature that state problems in understanding medical situations, confusion about their medications, trouble understanding medication labels, and bad reactions to medications due to language barriers.^23^ Compliance is related to poor health and treatment outcomes especially in patients with diabetes, epilepsy, hypertension, etc., and results in a financial burden for society.^24^

Threat to patient safety due to misdiagnosis and consequent treatment plan by the doctor as well as misinterpretation of treatment by the patient is also identified as one of the effects of the language barrier in our study and is supported by previous studies related to patient-doctor communication concerning diagnosis, risk communication, and acute situation as instances where patient safety was placed as high risk due to the presence of language barriers.^25^ Patients with language barriers were more likely to report problems in understanding how to use medication, had difficulty comprehending medication labels and were at risk of developing side effects to medication.^23^ Moreover, misinterpretation of patient needs, and medical issues further results in patient demotivation, poor treatment compliance, and sub-standard patient care.

### Limitations & Way Forward:

An adequate number of participants were interviewed in our study but they were limited to only two cities. That said, perspectives on the language barrier and its influence on the physician and patient relationship reached saturation. Further studies should be carried out in different cities to check the persistency of our results. Studies should also be conducted from the viewpoint of the patients that how language barriers influence their relationship with the physicians.

## CONCLUSION

This study establishes the detrimental effects of language barriers on the relationship between physician and patient which can help medical educationists and policymakers in devising a curriculum in such a way that it can minimize the impact of language barriers on the doctor-patient relationship. A course should be introduced at the undergraduate level that focuses on the translation of common signs and symptoms, patient complaints, and diseases into the local or national language. Teaching doctor-patient communication skills should also be formally incorporated into the medical school curriculum. Furthermore, physicians, health professionals, educators, and practice leaders can use this information to adapt and encourage behaviors that successfully address language barriers and avoid those that might contribute to disparities.

### Authors’ Contribution:

**RM** designed and developed the concept designed methodology, collected data, analyzed data, wrote and edited manuscript. RM was also responsible for maintaining the accuracy and integrity of the work.

**UM** designed and developed the concept designed methodology, analyzed data, edited all manuscript, edited the final manuscript.

**RAK** helped in developing the idea and methodology, edited all manuscript, edited the final manuscript.

**AA** helped in data collection, data analysis and edited final manuscript.

## References

[1] Pakistan Bureau of Statistics[Internet] Population by mother tongue.1998.

[2] The Constitution of the Islamic Republic of Pakistan [Internet] (1973). Constitution of the Islamic Republic of Pakistan.

[3] Pakistan Medical and Dental Council [Internet] (2011). Curriculum of MBBS.

[4] Junaid A, Rafi MS (2019). Communication barriers between doctors, nurses, and patients in medical consultations at hospitals of Lahore Pakistan. Pak Armed Forces Med J.

[5] Adnan M, Latif F, Abid S (2020). Communication barriers in Pakistan:Interpretative Phenomenological Analysis. Paradigms.

[6] Rehman A, Diah NM (2020). Managing Women's Matter:A cross-cultural study of Doctor-Patient relationship in Pakistan and Malaysia. Intellectual Discourse.

[7] De Moissac D, Bowen S (2019). Impact of language barriers on quality of care and patient safety for official language minority Francophones in Canada. J Patient Exp.

[8] Shoaib M, Ahmed SA, Mahmood SU, Hafiz MY (2016). Doctor patient language barrier –Compromising on quality care. J Ayub Med Coll Abbottabad.

[9] Jahan F, Siddiqui H (2019). Good communication between Doctor-Patient improves health outcome. Eur J Med Health Sci.

[10] Kavukcu N, Altıntaş KH (2019). The challenges of the healthcare providers in refugee settings:a systematic review. Prehosp Disaster Med.

[11] Timony PE, Gauthier AP, Serresse S, Goodale N, Prpic J (2016). Barriers to offering French language physician services in rural and northern Ontario. Rural Remote Health.

[12] Derose KP, Baker DW (2000). Limited English proficiency and Latinos'use of physician services. Med Care Res Rev.

[13] Ferguson WJ, Candib LM (2002). Culture, language, and the doctor-patient relationship. Fam Med Community Health.

[14] Diamond L, Izquierdo K, Canfield D, Matsoukas K, Gany F (2019). A systematic review of the impact of patient-physician non-English language concordance on quality of care and outcomes. J Gen Intern Med.

[15] Fields A, Abraham M, Gaughan J, Haines C, Hoehn KS (2016). Language matters:Race, trust, and outcomes in the pediatric emergency department. Pediatr Emerg Care.

[16] Molina RL, Kasper J (2019). The power of language-concordant care:a call to action for medical schools. BMC Med Educ.

[17] Guerrero N, Small AL, Schwei RJ, Jacobs EA (2018). Informing physician strategies to overcome language barriers in encounters with pediatric patients. Patient Educ Couns.

[18] Wei D, Xu A, Wu X (2020). The mediating effect of trust on the relationship between doctor-patient communication and patients'risk perception during treatment. Psych J.

[19] Huang IC, Du PL, Lin LS, Liu TY, Lin TF, Huang WC (2021). The Effect of Perceived Value, Trust, and Commitment on Patient Loyalty in Taiwan. Inquiry.

[20] Pryce H, Hall A, Marks E, Culhane BA, Swift S, Straus J, Shaw RL (2018). Shared decision?making in tinnitus care–An exploration of clinical encounters. Br J Health Psychol.

[21] Um KH, Lau AK (2018). Healthcare service failure:How dissatisfied patients respond to poor service quality. Int J Oper Prod Manag.

[22] Ng JH, Luk BH (2019). Patient satisfaction:Concept analysis in the healthcare context. Patient Educ Couns.

[23] Wilson E, Chen AH, Grumbach K, Wang F, Fernandez A (2005). Effects of limited English proficiency and physician language on healthcare comprehension. J Gen Intern Med.

[24] Jin J, Sklar GE, Oh VM, Li SC (2008). Factors affecting therapeutic compliance:A review from the patient's perspective. Ther Clin Risk Manag.

[25] van Rosse F, Bruijne de M, Suurmond J, Essink-Bot ML, Wagner C (2016). Language barriers and patient safety risks in hospital car:A mixed methods study. Int J Nurs Stud.

